# Walking biomechanics in women with patellofemoral osteoarthritis differ compared to men with and women without patellofemoral osteoarthritis

**DOI:** 10.1016/j.bjpt.2024.101132

**Published:** 2024-10-30

**Authors:** Matthew G King, David C Ackland, Harvi F Hart, Anthony G Schache, Prasanna Sritharan, Marcus G Pandy, Kay M Crossley

**Affiliations:** a Australian IOC Research Centre, La Trobe Sport and Exercise Medicine Research Centre, School of Allied Health, Human Services and Sport, La Trobe University, Bundoora, Victoria, Australia; b Department of Biomedical Engineering, University of Melbourne, Victoria, Australia; c School of Physical Therapy, Faculty of Health Sciences, Western University, Ontario, Canada; d Department of Mechanical Engineering, University of Melbourne, Victoria, Australia

**Keywords:** Gait, Knee, Patellofemoral osteoarthritis, Rehabilitation, Walking

## Abstract

**Background:**

Differences in walking biomechanics between women and men with patellofemoral joint (PF) osteoarthritis (OA) may contribute to the development or progression of persistent symptoms in people with PFJ OA.

**Objective:**

Evaluate how walking biomechanics of women with PF OA differ from: (i) men with PFJ OA; and (ii) women without PF OA. Second, explore the relationship between knee-related symptoms/function and walking biomechanics in individuals with PF OA, and whether these are modified by sex.

**Methods:**

Sixty-seven individuals with PF OA (43 women) and 14 women without PF OA were included. Biomechanics data were recorded during walking. Patient-reported symptoms and function were obtained using the Knee injury and Osteoarthritis Outcome Score. Differences in continuous biomechanical data were assessed using statistical parametric mapping, with discrete data and relationships evaluated using linear models.

**Results:**

Women with PF OA walked with a greater hip adduction angle throughout stance (*t* > 2.757) and lower impulses for the hip flexion, knee flexion, and ankle dorsiflexion moments (adjusted mean differences [95 % CI]:3.3 × 10^–2^ [-4.9 × 10^–2^, -1.6 × 10^–2^], -2.9 × 10^–2^ [-5.3 × 10^–2^, -0.4 × 10^–2^], -5.1 × 10^–2^ [-8.2 × 10^–2^, -2.0 × 10^–2^] Nms/kg, respectively) compared to men with PF OA. Compared to their asymptomatic peers, women with PF OA displayed a 5° offset towards greater hip flexion. Higher knee adduction moment impulse correlated with worse KOOS-ADL scores in men, not women.

**Conclusion:**

Observed biomechanical differences were small in nature with moderate to weak relationship observed with the KOOS. Findings were not limited to the knee, indicating that women with PF OA display unique biomechanical features across the kinetic-chain.

## Highlights

• Biomechanical impairments in patellofeomoral osteoarthritis are sex specific.
• Joint moment impulses of anti gravity muscles are lower in women with PF OA.
• Recommendations from male-dominated cohorts may not apply to women with PF OA.
• Sex as an effect modifier should be considered in future research.

## Introduction

Patellofemoral joint (PF) osteoarthritis (OA) is present in approximately one-half of individuals with knee pain or tibiofemoral joint (TF) OA.^1^ It is associated with considerable symptoms, functional limitations, and poor quality of life.^2^, ^3^, ^4^ Mechanical loading is thought to underpin the development and progression of OA,^5^ hence it is conceivable that altered lower-limb biomechanics during common activities of daily living, such as walking may play a role in the development and/or progression of PF OA.^6^^,^^7^

Studies have found features of PF OA to be sex-dependent. For example, women have a higher prevalence of isolated PF OA,^8^^,^^9^ and report more knee pain and disability than men.^10^ Sex differences have been observed in PF biomechanics in human cadavers,^11^ and in healthy participants during running.^12^ Moreover, biomechanical risk factors for PF pain development vary between women and men.^13^ These findings would suggest that sex is likely to influence the relationship between PF OA and walking biomechanics, consistent with what has been observed in people with TF OA,^14^^,^^15^ PF pain,^16^ and hip-related pain.^17^, ^18^, ^19^

Several studies have evaluated walking biomechanics in people with and without PF OA but have so far yielded mostly inconsistent results. Teng et al.^20^ found a higher peak knee flexion moment and impulse as well as peak PF pressure during the second half of stance in those with isolated PF OA compared with controls. Crossley et al.^21^ did not find any differences in knee and ankle joint angles during walking between individuals with and without PF OA, but found those with PF OA to display increased anterior pelvic tilt, contralateral pelvic drop, and hip adduction angles as well as a decreased hip extension angle. In contrast, Pohl et al.^22^ found no differences in contralateral pelvic drop, hip adduction, and hip internal rotation angles during walking between individuals with and without PF OA. Although two of these studies considered sex as a confounder within their analyses,^20^^,^^21^ none specifically explored sex as an effect modifier. If sex modifies the relationship between PF OA and walking biomechanics, it is possible that previous studies may have overlooked this effect, which could be one reason for the inconsistent findings. Further scrutiny regarding the influence of sex on the relationship between on PF OA and walking biomechanics is therefore warranted.

It is also possible that differences in walking biomechanics between women and men may influence the development or progression of structural joint changes and symptom persistency in people with PF OA. For example, women without clinical knee OA have greater PF cartilage volume loss over time compared with men,^23^ and a lower step rate is associated with greater worsening of PF cartilage damage in women but not in men.^24^ These sex-dependent findings, in conjunction with women with PF OA reporting greater knee pain severity,^10^^,^^25^ highlight the potential for the condition to affect men and women differently. With these points in mind, our study aims were twofold. First, to explore how the walking biomechanics of women with PF OA differ from: (i) men with PF OA; and (ii) women without PF OA. Second, to explore the relationship between walking biomechanics and knee-related symptoms/function in individuals with PF OA, and whether these are modified by sex. It was hypothesised that the walking biomechanics of women with PF OA would display both sex- and pathology-based effects and that sex would influence the relationship between walking biomechanics and knee-related symptoms/function in individuals with PF OA.

## Methods

### Design and participants

A cross-sectional design was employed using baseline data from a subset of individuals with PF OA from a previous randomized controlled trial.^26^ Individuals with PF OA were included if they were aged 40 years or over; had anterior – or retro-patellar pain severity of ≥4/10 during at least two PF loading activities on most days over the preceding month; and exhibited radiographic evidence of lateral PF OA (Kellgren and Lawrence^27^ Diagnostic Criteria [KL] ≥1^28,29^). Control women participants were concurrently recruited and eligible to participate if they were aged 40 years or over, had no knee or lower limb symptoms or pain, and had no radiographic evidence of TF or PF OA (i.e., KL<2).^28^ Exclusion criteria for all participants were: concomitant pain and/or symptoms from other knee structures, hips, or the lumbar spine; body mass index (BMI) ≥35kg/m^2^; previous lower limb osteotomy or arthroplasty; intra- or extra-articular knee joint injection in the preceding three months; radiographic TF OA (KL>2); unable to undertake study testing procedures; neurological or other conditions; and unable to understand spoken and written English. The study received ethical approval from the University of Melbourne Human Research Ethics Committee (HREC 0721163), and all participants provided written informed consent before participating.

### Radiographs and OA classification

To confirm eligibility, all participants underwent a semi-flexed, posteroanterior weight-bearing, and skyline radiograph that were graded by two trained individuals using the KL grading system^27^ which has been previously found to be reliable (κ 0.745 to 0.843).^29^^,^^30^

### Participant demographics and patient-reported outcome measures

Participant demographic data, including age, height, and body mass, were recorded, and individuals with PF OA completed the Knee Injury and Osteoarthritis Outcome Score (KOOS)^31^ prior to biomechanical testing. The KOOS evaluates the five subscales of Pain, Symptoms, Function in Daily Living (ADL), Function in Sport and Recreation (Sport/Rec), and Knee-related Quality of Life (QOL) over the previous week.^31^

### Biomechanical data collection

Biomechanical data collection was conducted at the The University of Melbourne. For testing, participants wore loose-fitting running shorts and Nike Straprunner sandals to allow adequate exposure of foot bony landmarks for marker placement. Opto-reflective markers were affixed to the participant's pelvis and lower limbs using a previously published protocol.^32^ Trajectories of the opto-reflective markers were recorded at 120 Hz using a nine-camera motion capture system (Vicon Motion Systems, UK), while ground reaction force (GRF) data were measured at 1080 Hz via three force platforms (AMTI, USA) embedded in the laboratory floor. All participants were required to complete at least three successful walking trials across a 10 m walkway through the laboratory's capture volume. A successful trial occurred when the participant achieved a single-foot contact in the middle of the force plate.

A seven-segment biomechanical model was used to calculate lower-limb joint angles and moments, with anatomical coordinate systems defined as previously outlined.^33^ A joint coordinate system convention was used to calculate lower-limb joint kinematics.^34^ Net joint moments were calculated using inverse dynamics, normalized to body mass (Netwon metres per kilogram; Nm/kg) and expressed in the same non-orthogonal joint coordinate system as the calculated hip, knee, and ankle joint angles. Each joint moment was reported as the external joint moment.^33^ We also calculated the impulse of each joint moment by taking the integral of the moment vs time curve across the stance phase of gait and expressed it as Newton metre seconds per kilogram (Nms/kg). The cumulative positive and negative impulses were calculated at each joint.

### Biomechanical variables of interest

To address the study's aims, sagittal and frontal plane kinematic (joint angles) and kinetic (joint moments and corresponding impulses) at the hip, knee, and ankle during the stance phase were selected as the biomechanical variables of interest. The impulse of the external joint moment provides insight into global loading experienced by passive (e.g., ligaments) and active (e.g., muscles) structures of the joint where both the magnitude and duration of the moment are taken into account,^18^ hence we selected the calculated impulses to test associations with KOOS subscales. Transverse plane data were not included as variables of interest due to their low reliability.^35^^,^^36^

### Data analysis

Demographic data and biomechanical variables of interest were assessed for normality and linearity using Shapiro-Wilk analyses as well as visual inspection of boxplots and Q-Q plots and reported as appropriate. Comparisons of continuous variables (joint angles and moments) between groups (women with vs men with PF OA; women with vs women without PF OA) were completed using two-sample *t*-tests via statistical parametric mapping (SPM) (spm1D v0.4.8, http://www.spm1d.org) conducted in python v3.8 (Python™, Python Software Foundation) using previously defined methods.^37^, ^38^, ^39^ In brief, the test statistic (SPMt) was calculated at each time node of the time series data and plotted.^39^ Alpha was set at 0.05 and the critical value of t was calculated based on the trajectory smoothness via temporal gradients.^37^^,^^38^ The differences between the two groups were considered meaningful when the SPMt surpassed the critical value (suprathreshold cluster). P-values for each suprathreshold cluster were then calculated via determining the probability that each cluster could have occurred from an equivalently smooth random process.^37^, ^38^, ^39^

Differences in the external joint moment impulse between groups were assessed using linear regression models via the "car" package with model regression assumptions verified using the “performance” package in R (R, R Foundation for Statistical Computing). For each impulse of interest, the independent variables of group and covariates of age and TF OA severity (KL score) were entered into the model with α set at 0.05. Adjusted mean differences and 95 % confidence intervals (95 % CI) were calculated, independent of significance level, using the "effects" package in R (R, R Foundation for Statistical Computing).

The relationships between the impulses of the external joint moments and KOOS subscales were also explored using linear models. For each impulse of interest, the independent variables of KOOS subscale, sex, age, and TF OA severity (KL score) were entered into the model along with a sex-by-KOOS interaction term. If the relationship between the impulse and KOOS subscale was modified by sex (interaction term *P* < 0.05), the data were stratified and separate linear models were conducted. Where the relationship was not modified by sex (interaction term *P* > 0.05), the interaction term was dropped, and the relationship between the impulse and KOOS subscale was assessed within the model, controlling for sex, age, and TF OA severity (KL score).

## Results

A total of 67 individuals with PF OA (43 women) and 14 women without PF OA were included in the study (Table 1). All three groups were of comparable age but were not homogenous regarding height and mass. Men with PF OA were taller (mean difference: 0.12 m; 95 % CI: 0.08, 0.15) and heavier (13.9 kg; 7.5, 20.2) than women with PF OA. Furthermore, women with PF OA were heavier than women without PF OA (8.9 kg; 2.0, 15.8). The KOOS-QOL was the lowest subscale for women with PF OA, whereas the KOOS-Sport/Rec was the lowest subscale for men with PF OA (Table 1).

**Table 1:** Demographic information and patient-reported outcome measures for men and women with patellofemoral joint osteoarthritis and women without patellofemoral joint osteoarthritis.

	Patellofemoral Joint Osteoarthritis		Controls
	*Men (n=24)*	*Women (n=43)*	Women (*n* = 14)
Age (years)	57 ± 12	55 ± 9	55 ± 7
Height (m)	1.76 ± 0.09	1.64 ± 0.06	1.63 ± 0.06
Mass (kg)	86 ± 14	72 ± 11	63 ± 11
BMI (kg/m^2^)	27.5 ± 3.7	26.5 ± 4.0	23.6 ± 3.7
Walking speed (m s^-1^)	1.37 ± 0.13	1.41 ± 0.14	1.35 ± 0.17
TF Joint OA K/L grade^a^			
0	9 (38 %)	14 (33 %)	10 (71 %)
1	6 (25 %)	12 (28 %)	4 (29 %)
2	9 (38 %)	17 (40 %)	0
PF Joint OA K/L grade^a^			
0	0	0	–
1	5 (21 %)	8 (19 %)	–
2	16 (67 %)	19 (44 %)	–
3	2 (8 %)	7 (16 %)	–
4	1 (4 %)	9 (21 %)	–
KOOS			
*Pain*	65 ± 14	64 ± 16	–
*Symptoms*	67 ± 18	62 ± 16	–
*Activities of daily living*	77 ± 14	71 ± 18	–
*Sports/recreation*	46 ± 23	44 ± 24	–
*Quality of life*	47 ± 12	41 ± 16	–

All data reported as means ± standard deviations unless indicated. a data reported as number (%).

BMI, body mass index; K/L, Kellgren and Lawrence; KOOS, Knee Injury and Osteoarthritis Outcome Score; OA, osteoarthritis; PF, patellofemoral; TF, tibiofemoral.

KOOS score range 0 – 100 (0 = worst symptoms and 100 = no symptoms).

### Walking biomechanics in women and men with PF OA

**Fig. 1:**
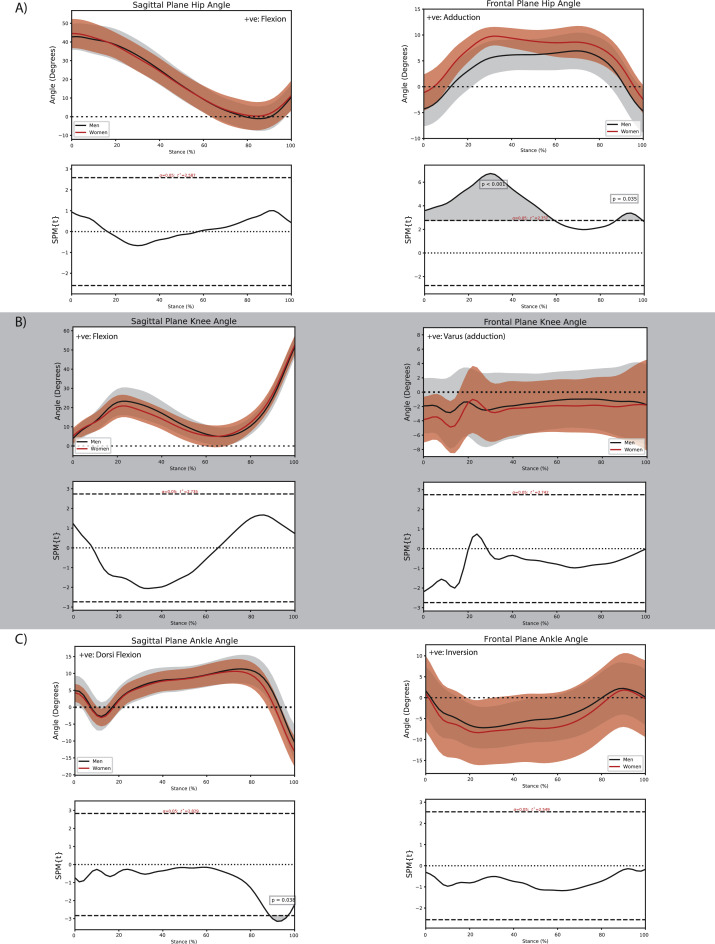
Comparison of sagittal and frontal plane kinematics between men and women with patellofemoral osteoarthritis at the A) hip, B) knee, and C) ankle during the stance phase of walking.

**Fig. 2:**
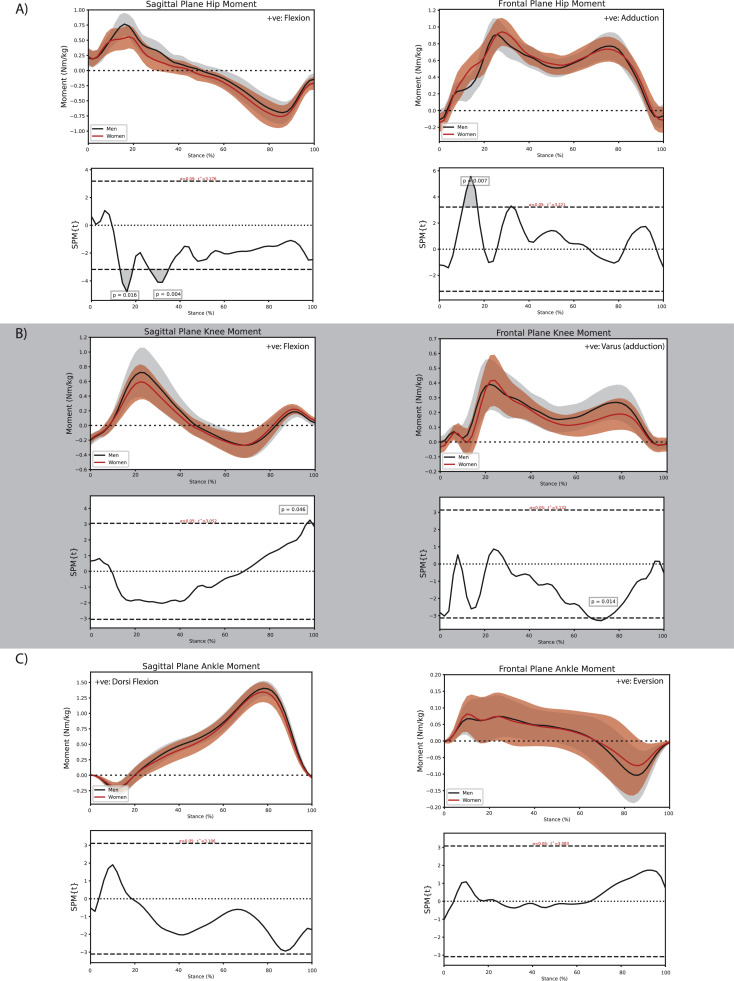
Comparison of sagittal and frontal plane external joint moments between men and women with patellofemoral osteoarthritis at the A) hip, B) knee, and C) ankle during the stance phase of walking.

Women and men with PF OA walked with comparable sagittal plane hip joint kinematics (Fig. 1A); however, women with PF OA demonstrated a smaller hip flexion moment (∼13 % to ∼19 % stance, *P* = 0.016; ∼26 % to ∼35 % stance, *P* = 0.004; Fig. 2A) and impulse (adjusted mean difference −3.3 × 10^–2^ (95 % CI −4.9 × 10^–2^, −1.6 × 10^–2^) Nms/kg, *P* < 0.01; Table 1) compared with men with PF OA. Women also demonstrated a greater hip adduction angle during early to midstance (initial contact to ∼59 % stance, *P* < 0.001; Fig. 1A) and late stance (∼88 % to toe-off, *P* = 0.035; Fig. 1A), in conjunction with a greater hip adduction moment during early stance (∼10 to ∼17 % stance, *P* = 0.007; Fig. 2A) compared with men with PF OA.

Women with PF OA walked with a smaller knee adduction moment during late stance (∼67 % to ∼74 % stance, *P* = 0.01; Fig. 2B) and demonstrated smaller impulses of the knee flexion and adduction moments (flexion: −2.9 × 10^–2^ (−5.3 × 10^–2^, −0.4 × 10^–2^) Nms/kg, *P* = 0.03; adduction: −3.0 × 10^–2^ (−5.3 × 10^–2^, −0.6 × 10^–2^) Nms/kg, *P* = 0.02; Table 1) compared with men with PF OA.

Women with PF OA walked with a greater ankle plantar flexion angle during late stance (∼89 % to ∼97 % stance, *P* = 0.038; Fig. 1C) and a smaller impulse of the ankle dorsiflexion moment (−5.1 × 10^–2^ (−8.2 × 10^–2^, −2.0 × 10^–2^) Nms/kg, *P* < 0.01; Supplementary material online 1) compared with men with PF OA.

### Walking biomechanics in women with and without PF OA

**Fig. 3:**
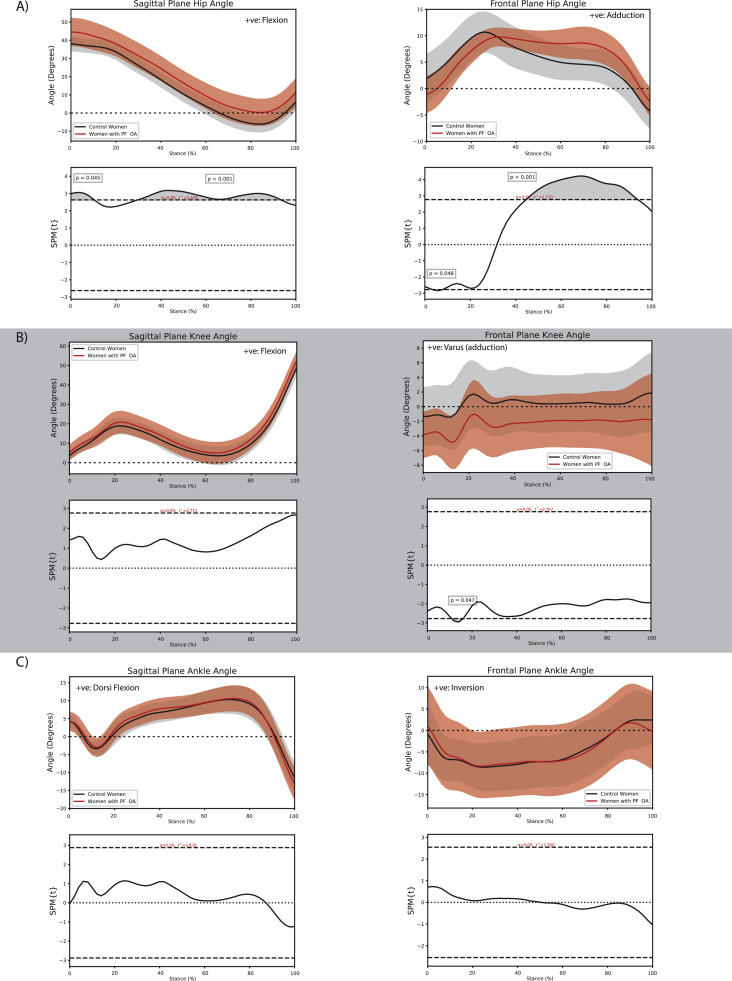
Comparison of sagittal and frontal plane kinematics between women with and without patellofemoral osteoarthritis at the A) hip, B) knee, and C) ankle during the stance phase of walking.

**Fig. 4:**
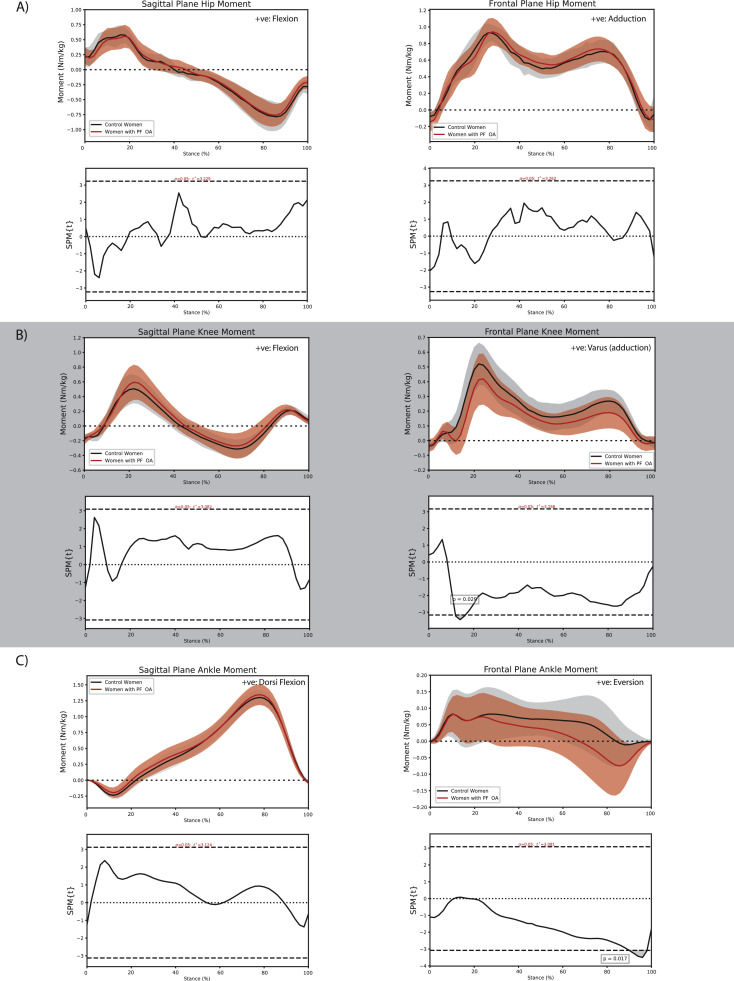
Comparison of sagittal and frontal plane external joint moments between women with and without patellofemoral osteoarthritis at the A) hip, B) knee, and C) ankle during the stance phase of walking.

Select differences in walking biomechanics between women with and without PF OA were observed across the lower-limb kinetic chain. At the hip, women with PF OA walked with a greater hip flexion angle throughout most of stance (initial contact to ∼10 % stance, *P* = 0.045; ∼30 % to 93 % stance, *P* < 0.001; Fig. 3A), a smaller hip adduction angle during early stance (∼3 % to ∼8 % stance, *P* = 0.048; Fig. 3A), and a larger hip adduction angle during mid to late stance (∼45 % to ∼94 % stance, *P* < 0.001; Fig. 3A). At the knee, women with PF OA walked with a greater knee valgus angle (∼11 % to ∼16 % stance, *P* = 0.047; Fig. 3B) but a smaller knee adduction moment during early stance (∼12 % to ∼16 % stance, *P* = 0.029; Fig. 4B) as well as a smaller impulse of the knee adduction moment (−4.1 × 10^–2^ (−6.9 × 10^–2^, −1.3 × 10^–2^) Nms/kg, *P* = 0.01; Supplementary material online 2). Finally, women with PF OA walked with a greater ankle inversion moment during late stance (∼90 % to ∼98 % stance, *P* = 0.017; Fig. 4C) compared with women without PF OA.

### External joint moment impulse and KOOS associations in men and women with PF OA

A sex-specific interaction was observed for the relationship between the impulse of the hip flexion moment and the KOOS-QOL (*F* = 4.48, *P* = 0.04; Supplementary material online 3). In men with PF OA, a lower impulse of the hip flexion moment was associated with worse scores for the KOOS-QOL subscale, whereas this relationship was not observed for women with PF OA (Supplementary material online 4A).

A positive relationship was observed between the impulse of the knee flexion moment and KOOS-Sport/Rec, independent of sex (Supplementary material online 3). Individuals with PF OA with worse scores for the KOOS-Sport/Rec subscale demonstrated a lower impulse of the knee flexion moment (Supplementary material online 4B).

Multiple relationships were observed between the impulse of the knee adduction moment and the KOOS. A sex-specific interaction was observed for the relationship between the impulse of the knee adduction moment and the KOOS-ADL (*F* = 4.75, *P* = 0.03; Supplementary material online 3). In men with PF OA, greater impulse of the knee adduction moment was associated with worse scores for the KOOS-ADL subscale, whereas this relationship was not observed for women with PF OA (Supplementary material online 4C). Furthermore, negative relationships were observed between the impulse of the knee adduction moment and the KOOS-Sport/Rec and KOOS-QOL, independent of sex (Supplementary material online 3). Individuals with PF OA with worse scores for the KOOS-Sport/Rec and KOOS-QOL subscales demonstrated a higher impulse of the knee adduction moment (Supplementary material online 4C).

## Discussion

The present study tested whether women with PF OA displayed differences in their walking biomechanics with respect to: (a) men with PF OA; and (b) women without PF OA. Overall, we found mostly similar joint angle and moment profiles between groups, apart from some select differences. We also identified some relationships between walking biomechanics and the KOOS for our cohort of people with PF OA. Interestingly, our findings were not limited to knee joint variables, indicating that women with PF OA display some unique biomechanical features across the entire lower-limb kinetic-chain.

We observed differences in walking biomechanics between women and men with PF OA. For example, women with PF OA walked with a larger hip adduction angle throughout most of the stance phase compared to men with PF OA. This result is consistent with what has been found by previous studies in healthy people^40^, ^41^, ^42^ and in those with knee OA.^41^ Such findings suggest that some fundamental differences in how women and men walk remain evident irrespective of joint pathology.^17^ We also found the impulses of the 'anti-gravity' external joint moments (i.e., external flexion moments) to be lower for women compared to men with PF OA. A similar outcome has been recently reported by Hart et al.,^43^ where women with PF OA walked with lower vertical ground forces and knee joint moments compared to men with PF OA. In contrast, other studies investigating sex-related differences in walking biomechanics for healthy people have in some instances, found women to display higher lower-limb joint moments than men.^40^^,^^42^^,^^44^ It therefore appears that sex-related differences in walking biomechanics are modified to some extent by the presence of joint pathology, and furthermore, some of our results might even be unique to people with PF OA.

We also observed some differences in walking biomechanics between women with PF OA when compared to sex- and age-matched controls. The most prominent differences were evident in kinematics at the hip rather than the knee. Women with PF OA were offset by ∼5° towards greater hip flexion throughout stance which is similar to previous findings of a forward trunk lean while walking^45^ and greater anterior pelvic tilt during stair ambulation.^46^ While this may be a movement strategy to lower overall vasti force and reduce PF loads,^21^^,^^45^^,^^46^ in our group the external knee flexion moment was not lower compared to controls. In the frontal plane, women with PF OA displayed greater hip adduction through mid to late stance, consistent with our previous publication that included all participants (women and men),^21^ without exploring any sex interactions. While women with PF OA walked with increased hip flexion and adduction angles, we did not observe any differences between groups in the corresponding hip moments in the same plane. Thus, hip joint loading remained similar between groups despite the differences in hip joint movement patterns. It is possible that the hip kinematic adaptations may have translated to differences in knee and ankle joint moments, highlighting the importance of evaluating the entire lower-limb kinetic chain.

Women with PF OA were offset by ∼2.5° towards knee valgus compared to women without PF OA. This offset likely resulted in the vertical GRF in the frontal plane being oriented closer to the knee joint centre, reducing the magnitude of its frontal plane lever arm about the knee joint centre, and thus the calculated external knee adduction impulse. Although we cannot determine causality, these findings may reflect an adaptive strategy to reduce frontal plane knee joint loading in the presence of pain and/or pathology.

Some moderate to weak associations were revealed between certain lower-limb joint moment impulses during walking and KOOS subscales, but it is worth noting that outcomes differed across anatomical planes in terms of whether higher or lower joint loading was more favorable. The two associations involving sagittal plane biomechanics variables indicated higher joint loading related to better subscales for the KOOS. In contrast, the three associations for the impulse of the knee adduction moment all indicated lower joint loading to be related to better subscales for the KOOS. Our findings here are generally consistent with some previous studies. In people with mild-to-moderate PF OA, Hart et al.^43^ found a higher impulse of the knee flexor moment during walking to be associated with a better (i.e., higher) KOOS. In people with medial TF OA, Hall et al.^47^ found associations between the knee adduction moment and symptoms to vary depending on disease severity. A lower impulse of the knee adduction moment was associated with less pain in those with moderate OA, whereas the opposite was true in those with severe OA. When interpreting these findings together, it seems that relationships between lower-limb joint mechanics and symptoms in people with knee OA are likely to be specific to both the dominant compartment involved and the disease severity.

There are limitations associated with the present study which require acknowledgement. First, the study's exploratory nature utilising a subset of participants from a previous randomized controlled trial^26^ means we did not perform an a priori sample size calculation. Consequently, the present study included groups with unbalanced numbers, which meant we may have been underpowered to detect additional differences or interaction effects resulting in type-II errors. Considering this and given the study's exploratory nature, we did not perform a statistical correction for multiple comparisons. Second, we did not constrain walking speed or control for it in our statistical analysis. Individuals were deliberately allowed to walk at their self-selected speed to ensure they replicated their natural/usual walking pattern in the laboratory setting.^48^ Lower limb walking biomechanics variables are sensitive to change with alterations in walking speed.^49^ Nevertheless, using a normalized/prescribed speed (or controlling for it within the statistical analysis) can potentially increase the likelihood for a type-II error.^50^^,^^51^ Third, we did not record the presence or severity of pain during the walking trials in the biomechanical assessment. Varying pain levels during walking may influence an individual's walking performance and should be considered when interpreting the results. Fourth, the imprecise diagnostic nature of PF OA, particularly the absence of a specific or sensitive clinical test to differentiate PF and TF OA,^21^ must be considered when interpreting the results. We believe the inclusion based on clinical findings and radiography provides reasonable assurance a PF OA dominant cohort was recruited. Finally, this study focused on lateral PF OA of generally mild severity, given its higher prevalence in PF OA populations.^1^^,^^29^ This limits the study's external validity, with results potentially not generalizable to everyone with PF OA.^21^

## Conclusion

Some select differences in walking biomechanics were evident when comparing women with PF OA to men with PF OA and to women without PF OA. Also, associations between walking biomechanics and symptoms in people with PF OA have the potential to be sex-specific. While some limited evidence was revealed in support of our hypothesis, further research is needed before definitive conclusions can be made. We suggest that evaluations combining male and female participants, without exploring the presence of sex as an effect modifier, may nullify associations between PF OA and walking biomechanics.

## Review board approval

Ethical approval was obtained from The University of Melbourne Human Research Ethics Committee (HREC 0721163)

## Contributions

MK, DA, and KC contributed to the conception and design of the study. AS, MP, and KC acquired the data. MK and PS analyzed the data with HH, AS, DA, and KC contributing to its interpretation. MK and HH drafted the manuscript with all authors contributing to its review and approval for submission.

## Conflicts of interest

The authors declare no competing interest.

## Appendix Supplementary materials

Image, application 1
