# Supplementary material for: Walking biomechanics in women with patellofemoral osteoarthritis differ compared to men with and women without patellofemoral osteoarthritis
Source: Braz J Phys Ther. 2024 Oct 30;28(6):101132. doi: 10.1016/j.bjpt.2024.101132 (PMC11564953; doi:10.1016/j.bjpt.2024.101132)

|  | Women with PF joint OA  *(n=43)*  Mean ± SD | Men with PF joint OA  *(n=24)*  Mean ± SD | *P* Value | Adjusted Mean Difference  (95% CI) |
| --- | --- | --- | --- | --- |
|  | *x 10^-2^*  Nms/kg | *x 10^-2^* Nms/kg |  | *x 10^-2^* Nms/kg |
| Hip Flexion Moment Impulse | 8.8 ± 3.0 | 12.2 ± 3.8 | <0.01* | -3.3 (-4.9, -1.6) |
| Hip Extension Moment Impulse | -14.4 ± 4.3 | -12.8 ± 4.2 | 0.21 | -1.3 (-3.4, 0.7) |
| Hip Adduction Moment Impulse | 35.7 ± 6.2 | 35.7 ± 7.2 | 0.79 | -0.5 (-3.7, 2.8) |
|  |  |  |  |  |
| Knee Flexion Moment Impulse | 10.0 ± 4.2 | 12.6 ± 6.3 | 0.03* | -2.9 (-5.3, -0.4) |
| Knee Extension Moment Impulse | -5.3 ± 3.0 | -5.8 ± 4.3 | 0.54 | 0.5 (-1.2, 2.3) |
| Knee Adduction Moment Impulse | 10.3 ± 4.0 | 13.2 ± 5.9 | 0.02* | -3.0 (-5.3, -0.6) |
|  |  |  |  |  |
| Ankle Dorsiflexion Moment Impulse | 34.5 ± 6.5 | 39.4 ± 6.0 | <0.01* | -5.1 (-8.2, -2.0) |
| Ankle Inversion Moment Impulse | 2.7 ± 2.2 | 2.6 ± 1.8 | 0.78 | 0.2 (-0.9, 1.2) |
| Ankle Eversion Moment Impulse | -1.5 ± 2.1 | -1.7 ± 1.7 | 0.61 | 0.3 (-0.7, 1.3) |
|  |  |  |  |  |

**Supplementary material online** **1:** Comparison of hip, knee, and ankle joint moment impulses between men and women with patellofemoral osteoarthritis during the stance phase of walking

* < 0.05, group means and standard deviations are reported unadjusted.

CI, confidence interval; OA, osteoarthritis; PF, patellofemoral; SD, standard deviation

|  | Women with PF joint OA  *(n=43)*  Mean ± SD | Women without PF joint OA  *(n=14)*  Mean ± SD | *P* Value | Adjusted Mean Difference  (95% CI) |
| --- | --- | --- | --- | --- |
|  | *x 10^-2^* Nms/kg | *x 10^-2^* Nms/kg |  | *x 10^-2^* Nms/kg |
| Hip Flexion Moment Impulse | 8.8 ± 3.0 | 9.3 ± 2.9 | 0.74 | -0.3 (-2.2, 1.5) |
| Hip Extension Moment Impulse | -14.4 ± 4.3 | -16.1 ± 3.5 | 0.13 | 2.1 (-0.3, 4.6) |
| Hip Adduction Moment Impulse | 35.7 ± 6.2 | 37.2 ± 11.0 | 0.53 | -1.6 (-25.5, 22.3) |
|  |  |  |  |  |
| Knee Flexion Moment Impulse | 10.0 ± 4.2 | 8.9 ± 3.4 | 0.66 | 0.6 (-1.8, 3.0) |
| Knee Extension Moment Impulse | -5.3 ± 3.0 | -6.2 ± 2.7 | 0.25 | 1.2 (-0.6, 3.0) |
| Knee Adduction Moment Impulse | 10.3 ± 4.0 | 15.0 ± 6.1 | 0.01* | -4.1 (-6.9, -1.3) |
|  |  |  |  |  |
| Ankle Dorsi Flexion Moment Impulse | 34.5 ± 6.5 | 34.8 ± 6.2 | 0.75 | -0.7 (-4.7, 3.3) |
| Ankle Inversion Moment Impulse | 2.7 ± 2.2 | 4.0 ± 2.9 | 0.10 | -1.3 (-2.8, 0.1) |
| Ankle Eversion Moment Impulse | -1.5 ± 2.1 | -0.6 ± 0.8 | 0.22 | -0.8 (-2.0, 0.4) |
|  |  |  |  |  |

**Supplementary material online** **2:** Comparison of hip, knee, and ankle joint moment impulses between women with and without patellofemoral osteoarthritis during the stance phase of walking

* < 0.05, group means and standard deviations are reported unadjusted.

CI, confidence interval; Nms/kg, Newton metre seconds per kilogram; OA, osteoarthritis; PF, patellofemoral; SD, standard deviation

**Supplementary material online** **3:** Linear model results evaluating the relationship between Knee Injury and Osteoarthritis Outcome Score and joint moment impulse during the stance phase of walking

| Dependent Variable | Independent Variable | Sex by KOOS Interaction | | KOOS | |
| --- | --- | --- | --- | --- | --- |
|  |  | F Value | *P-*Value | F Value | *P-*Value |
| Hip Flexion Moment Impulse | KOOS Pain | 1.13 | 0.29 | 0.38 | 0.54 |
|  | KOOS Symptoms | 0.00 | 0.99 | 0.51 | 0.48 |
|  | KOOS ADL | 1.30 | 0.26 | 0.58 | 0.45 |
|  | KOOS Sport/Rec | 1.22 | 0.27 | 0.38 | 0.54 |
|  | KOOS QOL | 4.48 | 0.04* | - | - |
|  |  |  |  |  |  |
| Hip Extension Moment Impulse | KOOS Pain | 2.03 | 0.16 | 0.21 | 0.65 |
|  | KOOS Symptoms | 0.56 | 0.46 | 1.07 | 0.31 |
|  | KOOS ADL | 0.20 | 0.65 | 0.34 | 0.56 |
|  | KOOS Sport/Rec | 0.28 | 0.60 | 1.01 | 0.32 |
|  | KOOS QOL | 2.68 | 0.11 | 0.57 | 0.45 |
|  |  |  |  |  |  |
| Hip Adduction Moment Impulse | KOOS Pain | 1.45 | 0.23 | 2.60 | 0.11 |
|  | KOOS Symptoms | 1.91 | 0.17 | 2.61 | 0.11 |
|  | KOOS ADL | 0.52 | 0.47 | 1.89 | 0.17 |
|  | KOOS Sport/Rec | 0.06 | 0.81 | 3.47 | 0.07 |
|  | KOOS QOL | 0.35 | 0.56 | 0.00 | 0.99 |
|  |  |  |  |  |  |
| Knee Flexion Moment Impulse | KOOS Pain | 0.29 | 0.59 | 1.78 | 0.33 |
|  | KOOS Symptoms | 0.09 | 0.77 | 0.97 | 0.33 |
|  | KOOS ADL | 0.98 | 0.32 | 2.20 | 0.14 |
|  | KOOS Sport/Rec | 1.26 | 0.27 | 5.98 | 0.02* |
|  | KOOS QOL | 0.49 | 0.49 | 3.04 | 0.09 |
|  |  |  |  |  |  |
| Knee Extension Moment Impulse | KOOS Pain | 0.02 | 0.90 | 2.65 | 0.11 |
|  | KOOS Symptoms | 0.88 | 0.35 | 2.36 | 0.13 |
|  | KOOS ADL | 0.95 | 0.33 | 0.65 | 0.42 |
|  | KOOS Sport/Rec | 0.28 | 0.60 | 1.01 | 0.32 |
|  | KOOS QOL | 0.06 | 0.80 | 1.21 | 0.28 |
|  |  |  |  |  |  |
| Knee Adduction Moment Impulse | KOOS Pain | 2.51 | 0.12 | 3.92 | 0.05 |
|  | KOOS Symptoms | 3.43 | 0.07 | 2.15 | 0.15 |
|  | KOOS ADL | 4.75 | 0.03* | - | - |
|  | KOOS Sport/Rec | 2.63 | 0.11 | 5.32 | 0.02* |
|  | KOOS QOL | 0.87 | 0.35 | 6.16 | 0.02* |
|  |  |  |  |  |  |
| Ankle Dorsiflexion Moment Impulse | KOOS Pain | 0.09 | 0.76 | 0.33 | 0.57 |
|  | KOOS Symptoms | 0.35 | 0.56 | 0.09 | 0.76 |
|  | KOOS ADL | 0.14 | 0.71 | 0.08 | 0.78 |
|  | KOOS Sport/Rec | 0.12 | 0.73 | 0.22 | 0.64 |
|  | KOOS QOL | 0.00 | 0.99 | 0.12 | 0.73 |
|  |  |  |  |  |  |
| Ankle Inversion Moment Impulse | KOOS Pain | 1.75 | 0.19 | 0.19 | 0.66 |
|  | KOOS Symptoms | 0.14 | 0.71 | 0.83 | 0.37 |
|  | KOOS ADL | 0.62 | 0.43 | 0.16 | 0.69 |
|  | KOOS Sport/Rec | 0.31 | 0.58 | 2.17 | 0.15 |
|  | KOOS QOL | 2.90 | 0.09 | 0.15 | 0.70 |
|  |  |  |  |  |  |
| Ankle Eversion Moment Impulse | KOOS Pain | 0.00 | 0.96 | 0.01 | 0.93 |
|  | KOOS Symptoms | 0.13 | 0.72 | 0.23 | 0.64 |
|  | KOOS ADL | 0.02 | 0.88 | 0.02 | 0.89 |
|  | KOOS Sport/Rec | 0.01 | 0.92 | 0.44 | 0.51 |
|  | KOOS QOL | 0.78 | 0.38 | 0.51 | 0.48 |
|  |  |  |  |  |  |

* P < 0.05

ADL, activities of daily living; KOOS, Knee Injury and Osteoarthritis Outcome Score; QOL, quality of life


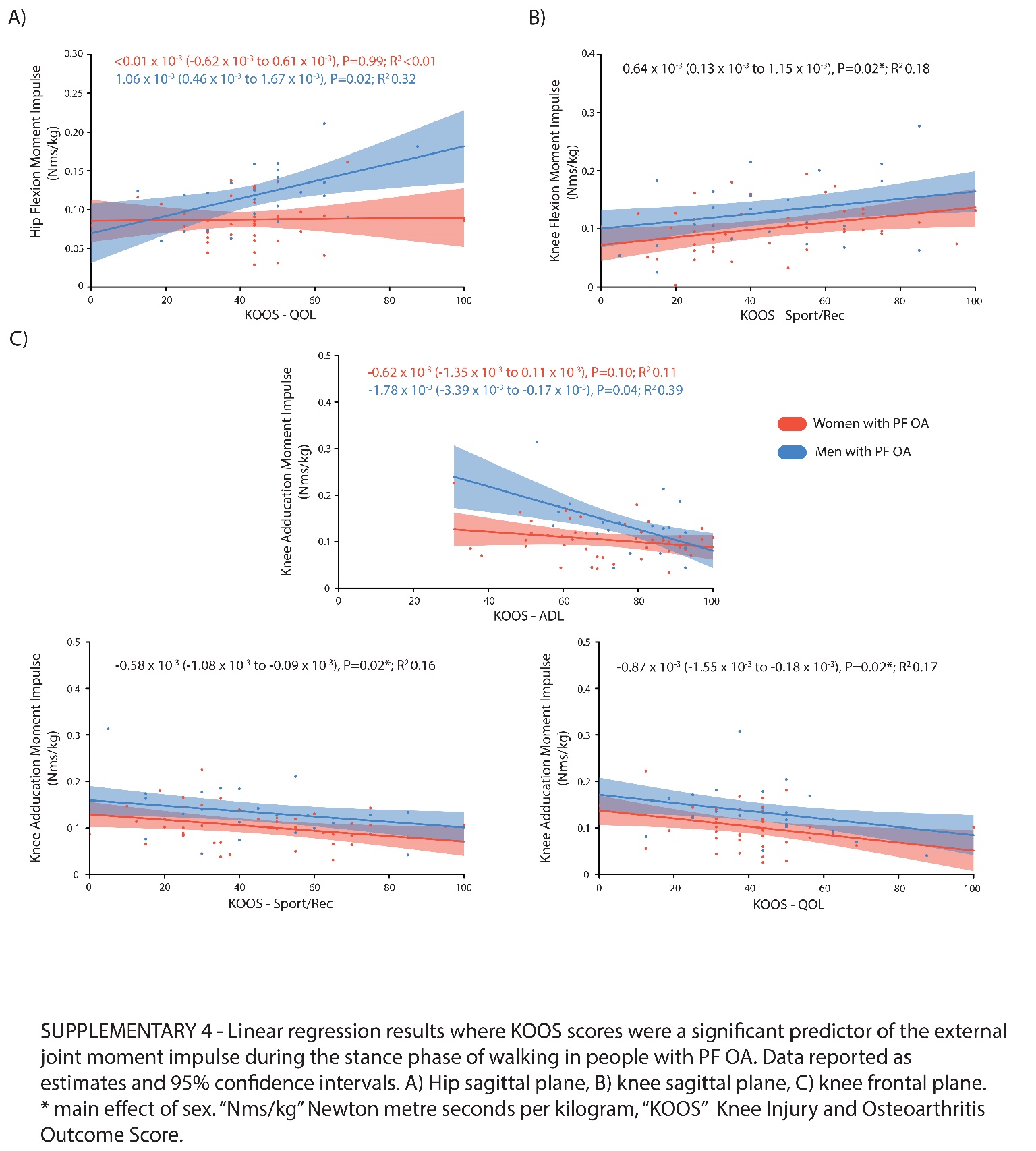

Supplement: Supplementary file 1 [file mmc1.docx]
